# NADPH-oxidase-driven oxygen radical production determines chondrocyte death and partly regulates metalloproteinase-mediated cartilage matrix degradation during interferon-γ-stimulated immune complex arthritis

**DOI:** 10.1186/ar1760

**Published:** 2005-05-20

**Authors:** Peter LEM van Lent, Karin CAM Nabbe, Arjen B Blom, Annet Sloetjes, Astrid EM Holthuysen, Jay Kolls, Fons AJ Van De Loo, Steven M Holland, Wim B Van Den Berg

**Affiliations:** 1 Department of Rheumatology, University Medical Centre, St Radboud, Nijmegen, The Netherlands; 2 Department of Pediatrics, University of Pittsburgh School of Medicine, Pittsburgh, PA, USA; 3 Department of Host Defenses, National Institute of Allergy and Infectious Diseases, Bethesda, MD, USA

## Abstract

In previous studies we have found that FcγRI determines chondrocyte death and matrix metalloproteinase (MMP)-mediated cartilage destruction during IFN-γ-regulated immune complex arthritis (ICA). Binding of immune complexes (ICs) to FcγRI leads to the prominent production of oxygen radicals. In the present study we investigated the contribution of NADPH-oxidase-driven oxygen radicals to cartilage destruction by using p47phox^-/- ^mice lacking a functional NADPH oxidase complex. Induction of a passive ICA in the knee joints of p47phox^-/- ^mice resulted in a significant elevation of joint inflammation at day 3 when compared with wild-type (WT) controls as studied by histology. However, when IFN-γ was overexpressed by injection of adenoviral IFN-γ in the knee joint before ICA induction, a similar influx of inflammatory cells was found at days 3 and 7, comprising mainly macrophages in both mouse strains. Proteoglycan depletion from the cartilage layers of the knee joints in both groups was similar at days 3 and 7. Aggrecan breakdown in cartilage caused by MMPs was further studied by immunolocalisation of MMP-mediated neoepitopes (VDIPEN). VDIPEN expression in the cartilage layers of arthritic knee joints was markedly lower (between 30 and 60%) in IFN-γ-stimulated arthritic p47phox^-/- ^mice at day 7 than in WT controls, despite significant upregulation of mRNA levels of various MMPs such as MMP-3, MMP-9, MMP-12 and MMP-13 in synovia and MMP-13 in cartilage layers as measured with quantitative RT-PCR. The latter observation suggests that oxygen radicals are involved in the activation of latent MMPs. Chondrocyte death, determined as the percentage of empty lacunae in articular cartilage, ranged between 20 and 60% at day 3 and between 30 and 80% at day 7 in WT mice, and was completely blocked in p47phox^-/- ^mice at both time points. FcγRI mRNA expression was significantly lower, and FcγRII and FcγRIII were higher, in p47phox^-/- ^mice than in controls. NADPH-oxidase-driven oxygen radical production determines chondrocyte death and aggravates MMP-mediated cartilage destruction during IFN-γ-stimulated IC-mediated arthritis. Upregulation of FcγRI by oxygen radicals may contribute to cartilage destruction.

## Introduction

During rheumatoid arthritis (RA), large numbers of inflammatory cells, mainly macrophages, migrate into the synovial layer [1]. Many of these macrophages become activated by mechanisms that are as yet unknown. Activated macrophages produce cytokines such as tumour necrosis factor-α (TNFα) and interleukin-1 (IL-1) and enzymes such as the metalloproteinase family, which can mediate severe cartilage destruction. A strong correlation was found between the number of activated macrophages and cartilage erosion [2]. Important triggers of macrophages are IgG-containing immune complexes, which are found in large amounts in the joints of many RA patients [3]. In previous studies we have found, by comparing various experimental arthritis models, that severe cartilage destruction developed mainly when immune complexes were present [4]. Severe cartilage destruction is thereby defined as chondrocyte death and cartilage matrix destruction. The latter is induced predominantly by metalloproteinases (MMPs), which are released in a latent form. Upon activation these enzymes degrade the collagen type II network in the cartilage resulting in irreversible erosion [5]. During immune complex (IC)-mediated arthritides, synovial macrophages seemed to be dominant factors in the induction of severe cartilage destruction [6].

IgG-containing ICs communicate with macrophages with FcγR. Three classes have been described, and previous studies in our laboratory showed that absence of the activating FcγRI and FcγRIII completely abrogated severe cartilage destruction [7-9].

The mechanism of FcγR-mediated chondrocyte death and MMP-mediated cartilage destruction is not known. However, we found recently that FcγRI is the dominant activating FcγR causing cartilage destruction [10,11]. In T cell-driven immune complex arthritis (ICA), chondrocyte death in FcγRI^-/- ^was completely abrogated, whereas MMP-mediated cartilage destruction was significantly diminished [12]. Moreover, ICA stimulated by local overexpression of the T cell factor IFN-γ showed pronounced chondrocyte death that was also completely mediated by FcγRI [13].

Binding of ICs to FcγRI causes intracellular signalling and triggers activation of the multicomponent enzyme NADPH oxidase, which catalyses the production of oxygen species [14]. The latter have been shown to be involved in cell death [15,16] and in the activation of metalloproteinases [17]. The active central role in NADPH oxidase is as the transmembrane cytochrome *b*_556_, which comprises two subunits, gp91phox and p22phox. p47phox is the cytosolic component of the NADPH oxidase complex that translocates to the membrane and associates with cytochrome *b*_556 _to form the active complex that catalyses the reduction of oxygen to superoxide. Functionally, p47phox increases the binding of p67phox to cytochrome *b*_556 _about 100-fold [18-20]. IFN-γ strongly stimulates p91 and also the expression of FcγRI. Binding of ICs to FcγRI further increases NADPH oxidase activity [21]. Phospholipase D-1 has been shown to be an important mediator between FcγRI signalling and the activation of NADPH oxidase [14,22]. The combination of IFN-γ and FcγRI stimulation might therefore result in a strong stimulation of NADPH oxidase, catalysing the production of large amounts of superoxide.

In the present study we investigated the effect of NADPH-oxidase-driven oxygen radicals in the generation of severe cartilage destruction during IFN-γ-accelerated ICA. For that purpose mice in which the p47phox gene had been knocked out were used; they are unable to form a functional NADPH oxidase complex [23] and are therefore unable to make oxygen species by the NADPH oxidase pathway. However, other oxygen-radical-producing pathways remain intact. We found that chondrocyte death was completely abrogated, whereas MMP-mediated cartilage destruction was significantly inhibited. FcγRI expression was significantly downregulated; in contrast, MMP gene expression in the synovium was higher, suggesting that oxygen radicals are involved in the activation step of MMPs.

## Materials and methods

### Animals

NADPH-oxidase-deficient (C57BL/6-p47phox^- /-^) mice were generated as described previously [23], and lack the cytosolic p47phox subunit of the NADPH oxidase multicomponent system. The knockout mice were backcrossed to the C57BL6 background for 15 generations; C57BL/6 mice (obtained from the Jackson Laboratory, Bar Harbor, ME, USA) were used as controls. In some experiments p47phox^- /- ^mice of intercross progeny (C57BL/6 × 129Sv) were used with their proper controls. Colonies were maintained at the National Institutes of Health (Bethesda, MD, USA). All mice were housed under specified pathogen-free conditions during breeding and experiments. Mice received autoclaved chow and acidified water *ad libitum*. Only healthy mice were used in the experiments and were age-matched (10 to 20 weeks) and sex-matched for each set of experiments. All experiments were approved by local authorities of the Animal Care and Use Committee (DEC 98.22) and performed by personnel certified by the Dutch Ministry of Well-being, Public Health and Culture.

### Overexpression of IFN-γ *in vivo *with an adenoviral construct

The recombinant adenovirus encoding murine IFN-γ (AdIFN-γ) was generated as described previously [24]. Knee joints of naive mice were injected intra-articularly with 6 μl of AdIFN-γ (10^7 ^plaque-forming units). At different time points (days 3 and 7), patellae with adjacent synovium were dissected in a standardised manner [25] and synovium biopsies were taken using a biopsy punch with a diameter of 3 mm. Total RNA was extracted in 1 ml of TRIzol reagent and used for quantitative PCR as described below. AdIFN-γ was injected intra-articularly 1 day before arthritis induction.

### Induction of immune complex arthritis

ICA was passively induced by injecting 3 μg of poly-(L-lysine)-coupled lysozyme into the knee joints of mice that had previously (16 hours earlier) received, intravenously, polyclonal antibodies directed against lysozyme. These antibodies were raised in rabbits.

### Histology of arthritic knee joints

Total knee joints of mice were isolated 3 and 7 days after arthritis onset. Mice were killed by cervical dislocation, knee joints were decalcified, dehydrated, and embedded in paraffin. Tissue sections (7 μm) were stained with haematoxylin and eosin. Seven sections spaced 70 μm apart representing the whole knee joint were measured to obtain a statistically justified result. Histopathological changes were scored by grading the inflammation on a scale from 0 (no inflammation) to 3 (severe inflamed joint) as the influx of inflammatory cells into synovium and joint cavity. To study proteoglycan (PG) depletion from cartilage matrix, sections were stained with safranin O followed by counterstaining with fast green. PG depletion (loss of red staining) from various cartilage layers was determined by using an arbitrary scale from 0 to 3. Normal cartilage was assigned the value 0, and cartilage fully depleted of PGs was taken as 3. Chondrocyte death was determined in total knee joint sections stained with haematoxylin and eosin. Chondrocyte death was determined as the percentage of the area of the cartilage containing empty lacunae in relation to the total area. All experiments were scored separately and independently from each other.

### Immunohistochemical detection of the identification marker of macrophages

F4/80, a murine macrophage membrane antigen, was detected with a specific rat anti-mouse F4/80 IgG. Primary antibodies were detected with rabbit anti-rat IgG and avidin–horseradish peroxidase conjugate. Finally, sections were counterstained with Mayer's haematoxylin (Merck, Darmstadt, Germany).

### Immunolocalisation of MMP-induced neoepitope (VDIPEN)

For immunohistochemical analysis of MMP-induced neoepitopes, sections were deparaffinised, rehydrated and digested with chondroitinase ABC (Sigma; 0.25 U/ml in 0.1 M Tris-HCl, pH 8.0) for 1 hour at 37°C, to remove chondroitin sulphate from the PGs. Sections were then treated for 20 min with 1% hydrogen peroxide in methanol and subsequently for 5 min with 0.1% (v/v) Triton X-100 in phosphate-buffered saline. After incubation for 20 min with 1.5% (v/v) normal goat serum, sections were incubated with affinity-purified anti-VDIPEN IgG overnight at 4°C. These antibodies were kindly provided by Irwin Singer and Ellen Bayne (Merck Research Laboratories, Rahway, NJ, USA) and have been extensively characterised previously [26,27]. In addition, sections were incubated with biotinylated goat anti-rabbit IgG and binding was detected by avidin-peroxidase staining (Elite kit; Vector Labs, Inc., Burlingame, CA, USA). Development of the peroxidase product was performed by nickel enhancement, and counterstaining was performed with Orange G (2%) for 5 min.

### Quantitative RT-PCR of synovium and cartilage

Synovial biopsies were taken from tissue adjacent to the suprapatellar ligament with a biopsy punch (diameter 3 mm). The cartilage layers from patellae and tibiae were isolated after decalcification with 5% EDTA for 4 hours at 4°C. Subsequently, patellae and tibiae were washed in 0.9% NaCl and the cartilage layer was carefully removed from the underlying bone with forceps and a dissection microscope. RNA was isolated with 1 ml of TRIzol reagent (Life Technologies, Breda, The Netherlands). Specific mRNA levels for various MMPs (MMP-2, MMP-3, MMP-9, MMP-12 and MMP-13), their inhibitors (TIMP-1, TIMP-2, TIMP-3 and TIMP-4) and FcγR (FcγRI, FcγRII and FcγRIII) were quantified with the ABI/PRISM 7000 Sequence Detection System (ABI/PE, Foster City, CA, USA). In brief, 1 μg of synovial RNA was used for RT-PCR. mRNA was reverse-transcribed to cDNA with the use of oligo(dT) primers; 1/20 of the cDNA was used in one PCR amplification. PCR was performed in SYBR Green Master Mix by using the following amplification protocol: 2 min at 50°C followed by 40 cycles of 15 s at 95°C and 1 min at 60°C, with data collection in the last 30 s. Message for murine glyceraldehyde-3-phosphate dehydrogenase, MMPs, MMP inhibitors and FcγR was amplified with specific primers (Biolegio, Malden, The Netherlands) for these molecules at a final concentration of 300 nM. Relative quantification of the PCR signals was performed by comparing the cycle threshold value (*Ct*) of the various molecules in the different samples after correction of the glyceraldehyde-3-phosphate dehydrogenase content for each individual sample to rule out confounding by variation of the RNA purification and reverse transcriptase steps.

## Results

### During ICA, joint inflammation is downregulated by oxygen radicals, which is compensated for by IFN-γ

**Figure 1:**
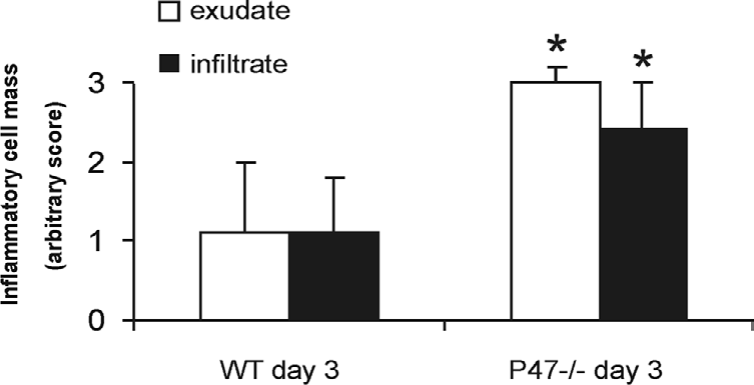
Knee joints, 3 days after induction of ICA in p47phox^-/- ^mice and their WT controls. The numbers of cells present in the synovium (infiltrate) and in the joint cavity (exudate) were determined on an arbitrary scale from 0 to 3: 0, no cells; 1, few; 2, moderate; 3, maximal. The number of cells was determined by two blinded observers. Data are means ± SD for eight animals. Significance was tested with the Wilcoxon rank test (*P < 0.05).

To investigate the effect of the NADPH-oxidase-driven production of oxygen radicals on joint inflammation, ICA was induced in knee joints of p47phox^-/- ^mice and their wild-type (WT) controls. Total knee joint sections were stained with haematoxylin and eosin and the numbers of inflammatory cells present within the synovium (infiltrate) and joint cavity (exudate) were determined by using an arbitrary scale of 0 to 3. At day 3 after the induction of ICA, joint inflammation was significantly higher in p47phox^-/- ^mice than in their WT controls (Fig. 1), indicating that oxygen radicals inhibit IC-mediated joint inflammation.

**Figure 2:**
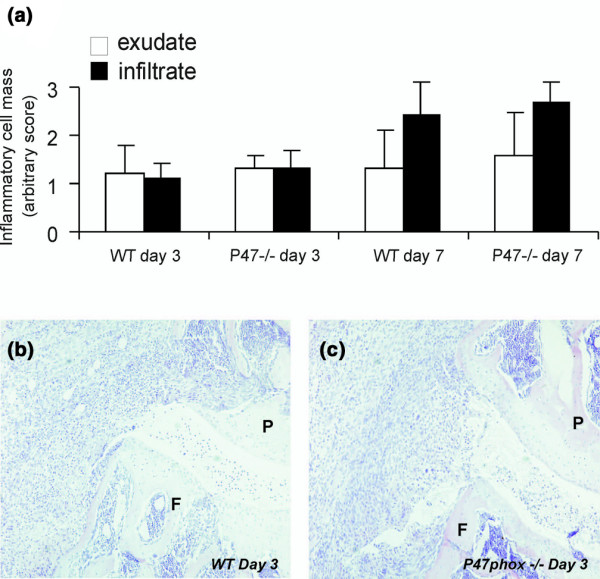
Inflamed knee joints, various days after IFN-γ-stimulated ICA in p47phox^-/- ^mice and controls. The numbers of cells present in the synovium (infiltrate) and in the joint cavity (exudate) were determined on an arbitrary scale from 0 to 3: 0, no cells; 1, few; 2, moderate; 3, maximal. **(a) **Numbers of cells; **(b) **wild-type controls; **(c) **p47phox^-/- ^mice. The number of cells was determined by two blinded observers. Data are means ± SD for eight animals. Significance was tested with the Wilcoxon rank test (*P < 0.05). Original magnifications × 100. F, femur; P, patella. Note that there is a comparable cell mass in arthritic knee joints of p47phox^-/- ^mice and wild-type controls.

In addition, the effect of IFN-γ on joint inflammation was investigated by injecting an adenoviral IFN-γ construct into the knee joints of p47phox^-/- ^mice and their WT controls, one day before ICA induction. Previous studies had shown that IFN-γ does not elevate joint inflammation during ICA, whereas cartilage destruction is significantly enhanced [13]. The latter is strongly correlated with upregulation of FcγRI. We find that the amount of inflammatory mass was comparable in IFN-γ-stimulated knee joints of p47phox^-/- ^mice and in their WT controls at both day 3 and day 7 after ICA induction (Fig. 2), suggesting that IFN-γ compensates for the aggravating effect of oxygen radicals on joint inflammation.

**Figure 3:**
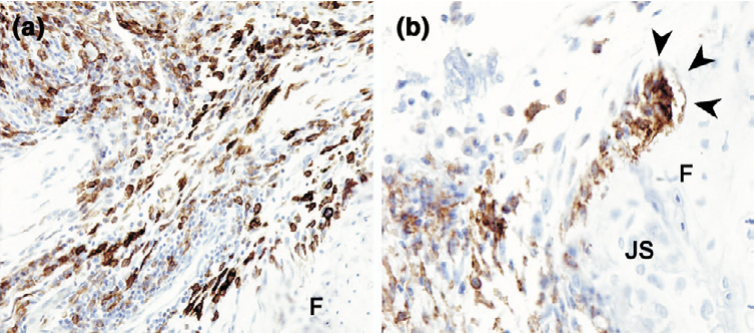
Expression of macrophage marker F4/80 in knee joints of day 7 IFN-γ-stimulated ICA. Note that 70 to 80% of the infiltrated cells within the synovium consist of macrophages **(a)**. Arrowheads in **(b) **indicate F4/80-positive macrophages attached to the cartilage surface and found in the lacunae of erosion pits. Original magnification × 400. F, femur; JS, joint space.

Apart from the amount, the cell type of the infiltrated cells might also be different. To determine the contribution of macrophages, the dominant cell type involved in cartilage destruction within this model, sections were stained with antibodies directed against F4/80. At day 7 after IFN-γ-stimulated ICA induction, high but comparable amounts of F4/80-positive macrophages were detected in both p47phox^-/- ^mice and their WT controls. Between 70 and 80% of the inflammatory cells, in both infiltrate and exudate, showed clear F4/80 staining (Fig. 3a). Moreover, large amounts of F4/80-positive macrophages were attached to cartilage surfaces at sites where erosion was detected (Fig. 3b).

### Oxygen radicals are not involved in mediating early PG depletion

**Figure 4:**
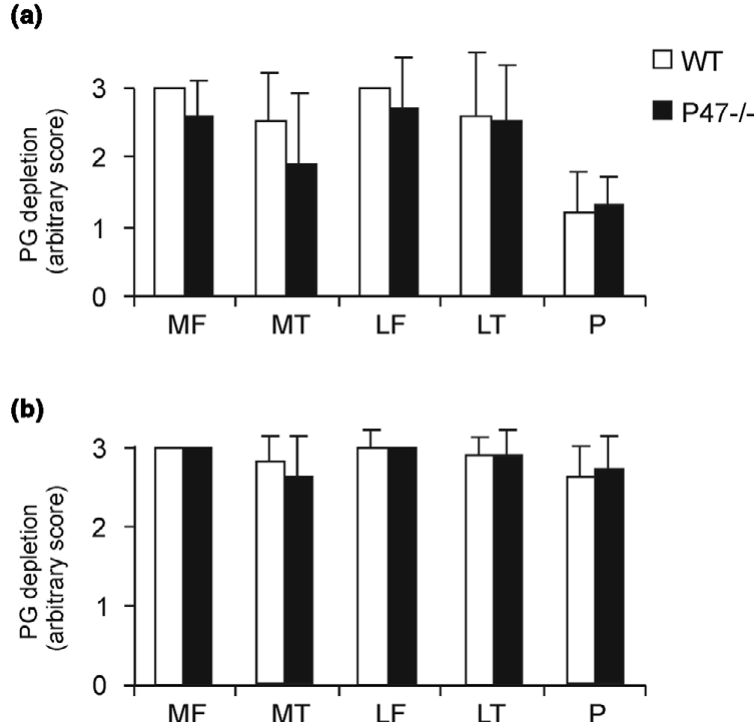
Proteoglycan loss from various cartilage layers of inflamed knee joints. Loss of red staining was scored in tibia, femur and patella on an arbitrary scale from 0 to 3. Data are expressed as loss of red staining in comparison with control cartilage layers, and are means ± SD for eight mice; they were tested for significance with the Wilcoxon rank test (*P < 0.05). No significant difference in proteoglycan loss was found on day 3 **(a) **or day 7 **(b) **between wild-type controls and p47phox^-/- ^mice. LF, lateral femur; LT, lateral tibia; MF, medial femur; MT, medial tibia; P, patella.

During ICA, mild cartilage destruction starts with the release of PGs from the surface of the cartilaginous layers. To investigate this early cartilage destruction, which is mainly mediated by aggrecanases, total knee joint sections were stained with safranin-O. Loss of red staining (a measure of PG loss), was scored on an arbitrary scale from 0 to 3 in various cartilage layers of the knee joint (medial and lateral femur, tibia and patella). At day 3 after IFN-γ-stimulated arthritis induction, PG loss in arthritic WT controls varied from 1 in the patella to 3 in the lateral and medial femur. At day 7 after ICA induction, nearly maximal PG loss was found in all cartilage layers investigated. Comparable PG depletion was found in arthritic p47phox^-/- ^knee joints at both day 3 and day 7 after arthritis induction (compare Fig. 4a with Fig. 4b), suggesting that NADPH-oxidase-driven oxygen radicals do not alter the aggrecanase activity responsible for PG loss. Arthritic knee joints not previously stimulated by IFN-γ also showed maximal PG loss that was not different between the two strains (data not shown).

### Oxygen radicals aggravate MMP-mediated cartilage destruction during IFN-γ-accelerated ICA

**Figure 5:**
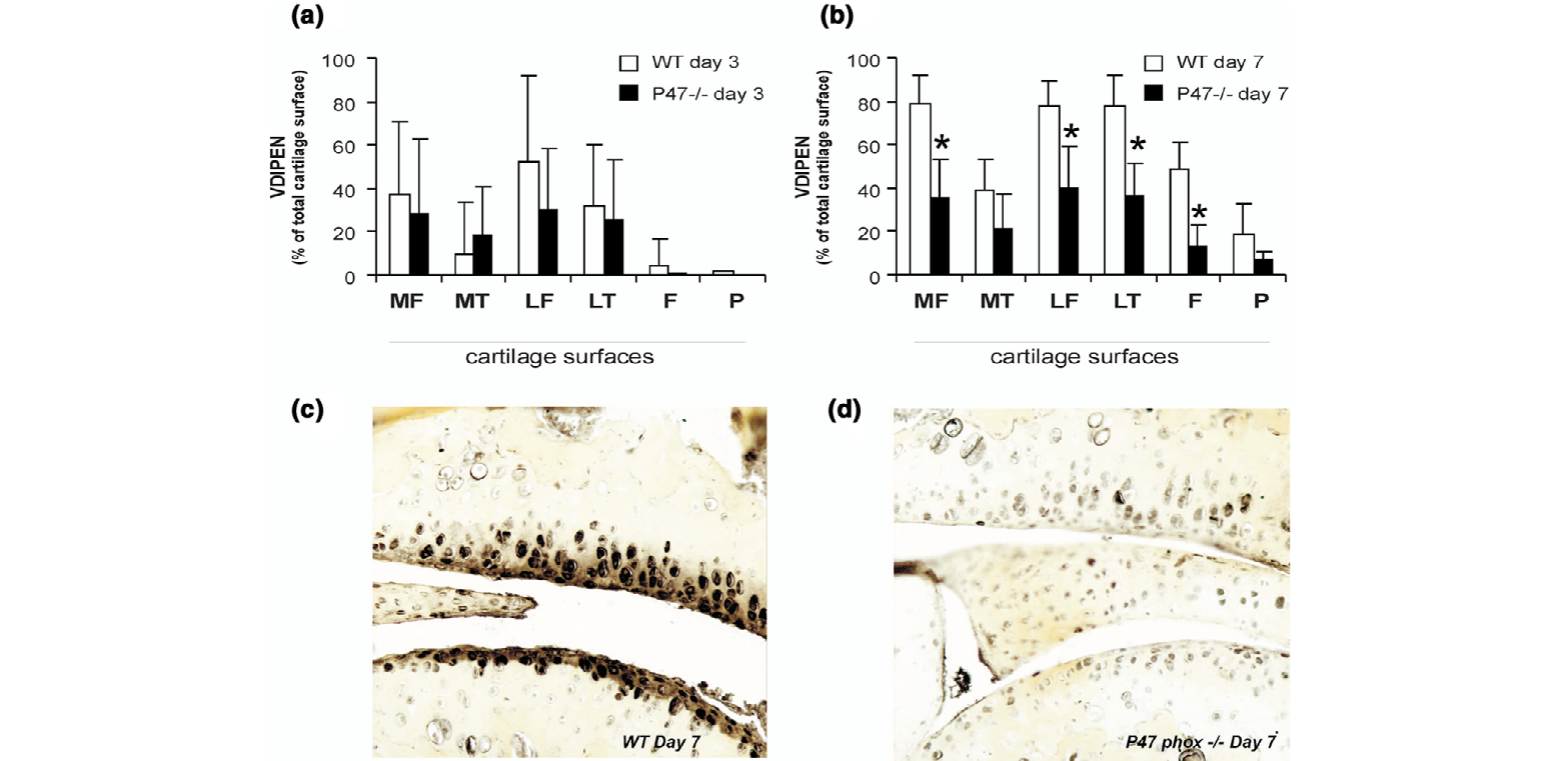
VDIPEN expression inday 3 **(a) **and day 7 **(b) **inflamed knee joints. Positive VDIPEN staining was determined in various cartilage layers (LF, lateral femur; LT, lateral tibia; MF, medial femur; MT, medial tibia; P, patella) at an original magnification of ×100 by using automated image analysis and was expressed as a percentage of the total cartilage surface. VDIPEN staining was significantly lower at day 7 in lateral and medial femur and lateral tibia of p47phox^-/- ^mice **(d) **than in wild-type mice on day 7 **(c) **Data are means ± SD for eight mice.

Because PG loss was not different between p47phox^-/- ^mice and WT controls, we additionally investigated the more severe cartilage matrix destruction mediated by MMPs. For this purpose, the amount of MMP-specific neoepitope VDIPEN expressed within various cartilage layers within the knee joint was determined by immunostaining with specific anti-VDIPEN antibodies. A progressive amount of VDIPEN staining was observed at day 7 when compared with day 3 in cartilage layers of IFN-γ-stimulated arthritic knee joints but not in p47phox^-/- ^mice (compare Fig. 5a with Fig. 5b).

In WT controls, the amount of VDIPEN staining varied from 5% in the patella to 55% in the lateral femur 3 days after arthritis induction. In p47phox^-/- ^mice, VDIPEN staining in various cartilage layers was comparable to WT controls at that time point (Fig. 5a). At day 7 after arthritis induction, VDIPEN staining varied between 10 and 80% in WT controls. Interestingly, in knee joints of arthritic p47phox^-/- ^mice, VDIPEN staining was significantly lower in the lateral femur, medial femur and lateral tibia (50%, 60% and 50% reduction, respectively) (Fig. 5b, and compare Fig. 5c with Fig. 5d) and values were not different from those found at day 3. These results indicate that oxygen radicals aggravate MMP-mediated cartilage damage during IC-mediated arthritis.

### Oxygen radicals downregulate MMP mRNA levels within cartilage layers and inflamed synovium during ICA

**Figure 6:**
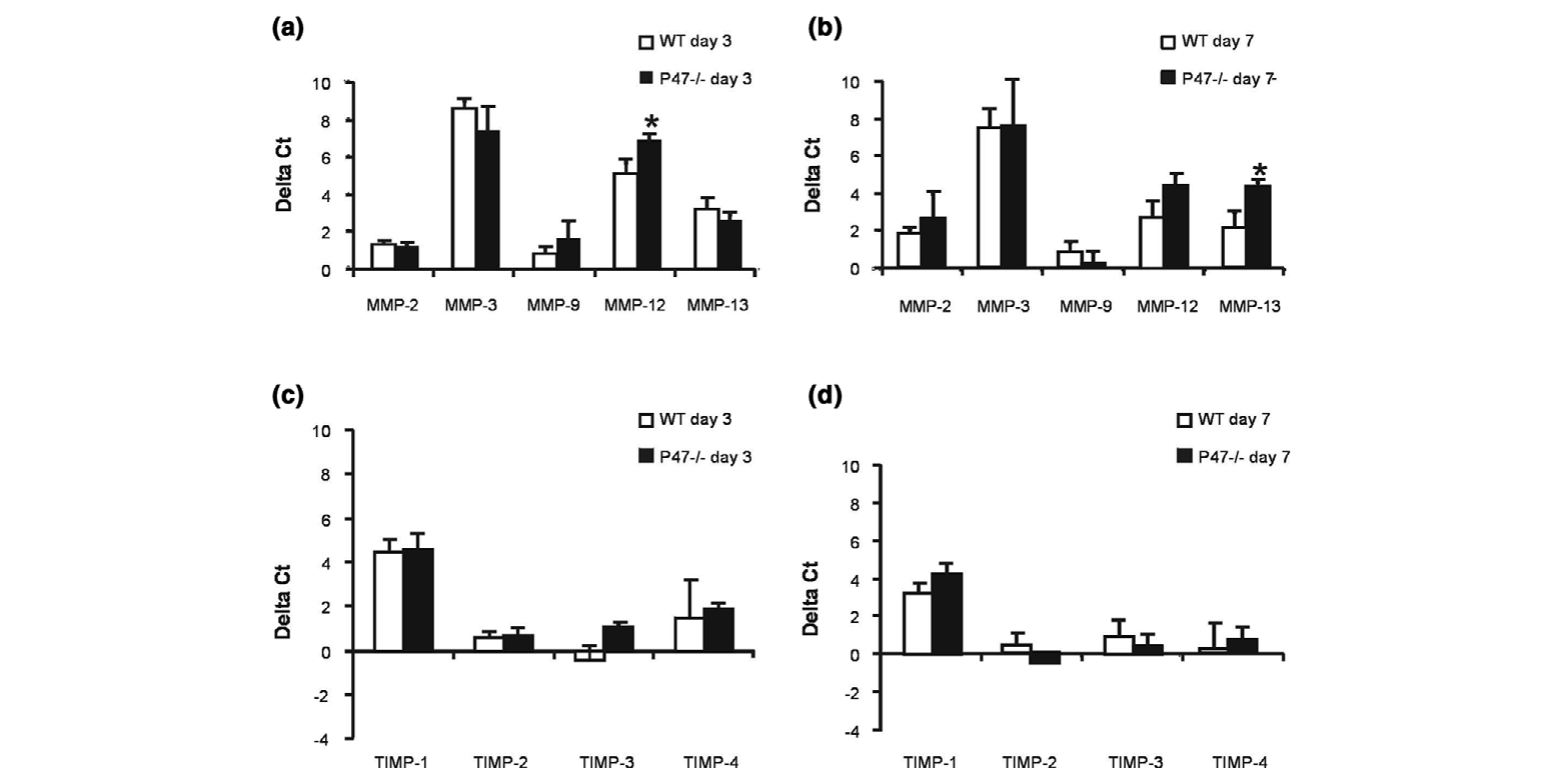
mRNA levels of various MMPs and TIMPs in cartilage layers derived from inflamed knee joints. Cartilage layers were isolated from patellae and tibia, three and seven days after IFN-γ accelerated arthritis. The cycle threshold value (Ct) of the various MMP and TIMP genes was corrected for glyceraldehyde-3-phosphate dehydrogenase content and *t *= 0. Note that at day 3, MMP-12 and at day 7 MMP-3 mRNA levels were significantly elevated in p47phox^-/- ^mice when compared to controls (6A and B). TIMP mRNA levels were not altered (6C and D).

One important source of MMPs involved in cartilage destruction might be derived from activated chondrocytes. To investigate whether oxygen radicals alter the expression of MMPs within the cartilage of an inflamed knee joint, patellar and tibial cartilage layers were isolated at day 3 and day 7 after arthritis induction, and mRNA levels of various MMPs (MMP-2, MMP-3, MMP-9, MMP-12 and MMP-13) and their inhibitors (TIMP-1, TIMP-2, TIMP-3 and TIMP-4) were determined by quantitative RT-PCR. IFN-γ-stimulated arthritis induced a marked increase in MMP-3, MMP-12 and MMP-13 mRNA levels in the cartilage layers of both WT controls and p47phox^-/- ^mice, in comparison with the cartilage of naive knee joints (Δ *Ct *ranging from 3 to 9) (Fig. 6a,b). However, MMP-12 and MMP-13 levels were significantly increased in the cartilage of arthritic p47phox^-/- ^knee joints at day 3 and day 7, respectively, in comparison with WT controls (Fig. 6a,b). TIMP-1 was the only inhibitor moderately expressed in cartilage after the induction of IFN-γ-stimulated arthritis, and no differences were observed between arthritic knee joints of WT controls and those of p47phox^-/- ^mice (Fig. 6c,d).

**Figure 7:**
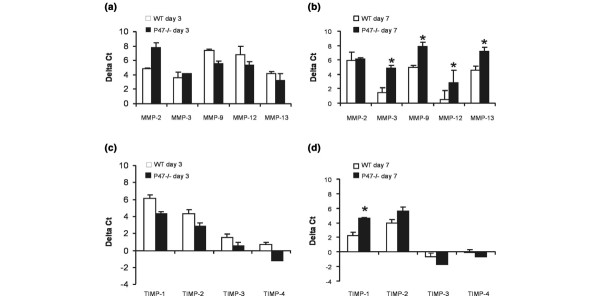
mRNA levels of various MMPs and TIMPs in synovia derived from inflamed knee joints. Inflamed synovia were isolated 3 days **(a,c) **and 7 days **(b,d) **after IFN-γ-accelerated arthritis. The cycle threshold value (*Ct*) of the various MMP **(a,b) **and TIMP **(c,d) **genes was corrected for glyceraldehyde-3-phosphate dehydrogenase content and for values at zero time. Note that at day 7 significantly elevated levels of MMP-3, MMP-9 and MMP-13 were found in p47phox^-/- ^mice in comparison with controls (b). TIMP-1 and 2 were also somewhat elevated, although to a lesser extent than MMP (d). No significant differences were found at day 3 (a,c).

Another important source of MMPs might be the inflamed synovium. Well-defined synovial specimens were isolated at days 3 and 7 after arthritis induction. At days 3 and 7 after IFN-γ accelerated ICA, MMP mRNA levels were evidently present in the cartilage of WT controls and p47phox^-/- ^mice, when compared with naive knee joints (Fig. 7a,b). Interestingly, at day 7, the cartilage of p47phox^-/- ^mice showed a significant elevation of MMP-3, MMP-9 and MMP-13 in comparison with that of WT controls (Fig. 7b). The expression of TIMP mRNA was determined in WT controls and p47phox^-/- ^mice: TIMP-1 and TIMP-2 were moderately expressed after the induction of arthritis in both groups (Fig. 7c,d). Moreover, the expression of TIMP-1 at day 7 after arthritis onset was significantly higher in p47phox^-/- ^mice than in the WT controls (Fig. 7d). Our data suggest that MMP mRNA levels are higher, and are certainly not decreased, in the cartilage layers and synovium of inflamed knee joints of p47phox^-/- ^mice.

### Oxygen radicals upregulate FcγRI and downregulate FcγRII and FcγRIII during IFN-γ-stimulated ICA

**Figure 8:**
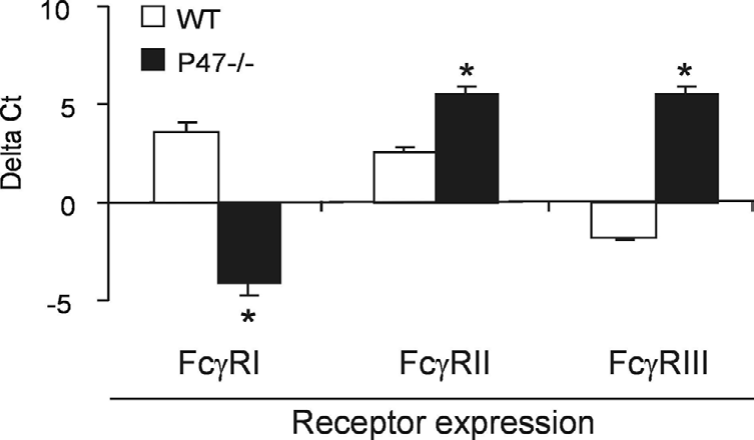
mRNA levels of various FcγRs in synovia derived from inflamed knee joints. Inflamed synovia were isolated 7 days after IFN-γ-stimulated arthritis. The cycle threshold value (*Ct*) of the various FcγR genes was corrected for glyceraldehyde-3-phosphate dehydrogenase content and for values at zero time. Note that at day 7 significantly lower levels of FcγRI and elevated levels of FcγRII and FcγRIII were found in p47phox^-/- ^mice than in controls.

In previous studies we found that activating FcγR (mainly FcγRI), predominantly expressed by haemopoietic cells present in the synovium, are important in the activation step of latent MMPs [10,11]. To investigate further whether oxygen radicals are involved in the regulation of FcγR, mRNA levels of the three FcγR classes were determined in synovia of day 7 IFN-γ-stimulated ICA. In WT mice, FcγRI and FcγRII were still upregulated 16 and 4 times, respectively whereas the expression of FcγRIII was four times lower than at zero time. In p47phox^-/- ^mice, FcγRI was downregulated (four cycli), whereas FcγRII and FcγRIII were both strongly upregulated (difference 64 times from zero time in both strains; Fig. 8).

### Oxygen radicals determine chondrocyte death during IFN-γ-driven IC-mediated arthritis

**Figure 9:**
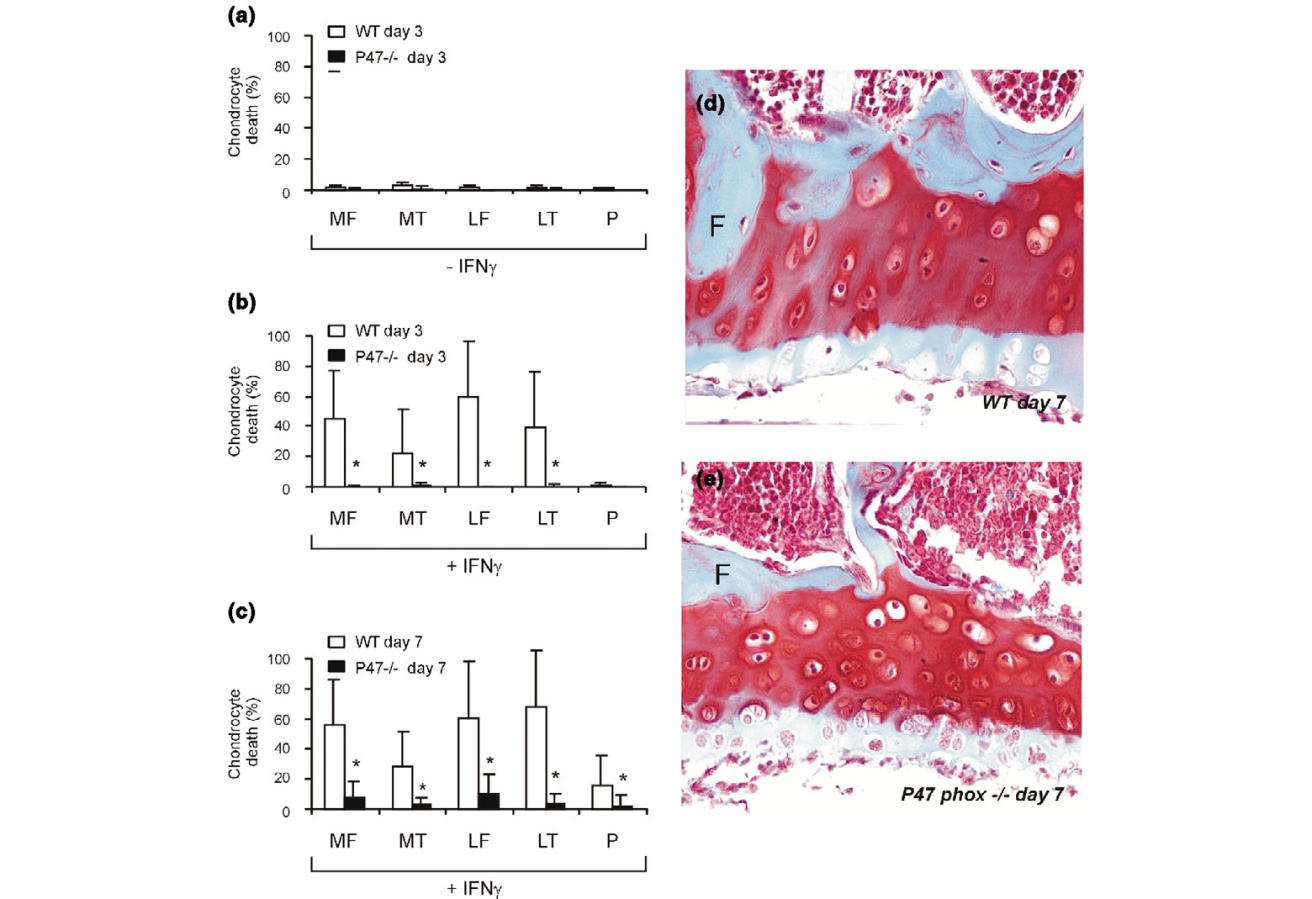
Chondrocyte death in cartilage layers of inflamed knee joints of p47phox^-/- ^and wild-type mice). At day 3 after ICA induction **(a) **and at day 3 **(b) **and day 7 **(c) **after IFNγ-stimulated ICA. Chondrocyte death was determined in various cartilage layers of the knee joint (LF, lateral femur; LT, lateral tibia; MF, medial femur; MT, medial tibia; P, patella). Chondrocyte death was expressed as a percentage of empty lacunae. Note that without IFN-γ no chondrocytes were observed. At day 7 after IFNγ-stimulated ICA, chondrocyte death was clearly present in wild-type controls **(d)**, whereas in p47phox^-/- ^mice chondrocyte death was completely absent **(e)**. Original magnifications ×400.

Apart from MMP-mediated cartilage destruction, chondrocyte death is an important parameter of severe cartilage destruction. In earlier studies we found that during IFN-γ-accelerated ICA, chondrocyte death was completely dependent on FcγRI. Because the binding of ICs to FcγRI results in the substantial production of oxygen radicals [14] and subsequently leads to a significant upregulation of this receptor, we further investigated whether NADPH-oxidase-driven oxygen radical production is indeed responsible for chondrocyte death in this model. The numbers of empty lacunae (resulting from chondrocyte death) present within various cartilage layers of the knee joint were determined and expressed as a percentage of the total numbers of chondrocytes present. Without IFN-γ overexpression, ICA did not induce chondrocyte death in normal WT and p47phox^-/- ^knee joints (Fig. 9a). In contrast, when AdIFN-γ was injected before arthritis induction, chondrocyte death increased tremendously and varied between 40 and 60% in the lateral and medial femur and between 20 and 40% in the lateral and medial tibia (Fig. 9b) at day 3 in WT mice. At day 7, chondrocyte death was even higher (between 60 and 70% in the femur and between 20 and 70% in the tibia; Fig. 9c,d). Interestingly, in arthritic knee joints of IFN-γ-stimulated p47phox^-/- ^mice, although joint inflammation was comparable to that found in WT mice, chondrocyte death was completely absent at day 3 and was only very low at day 7 (between 2 and 5% in the tibia and between 5 and 8% in the femur; Fig. 9b,c,e). Chondrocyte death does lead to cartilage erosion. However, at day 7 after IFN-γ-stimulated arthritis induction, erosion was still mild in knee joint cartilage layers of arthritic WT mice. Erosion pits were found only in the superficial layers of the medial and lateral tibia. Clear attachment of macrophages to the cartilage surface was observed. Cartilage layers in the knee joints of arthritic p47phox^-/- ^mice showed similar attachment of macrophages and only mild erosion, whereas no chondrocyte death was observed (Fig. 9e).

## Discussion

In the present study we found that in the absence of NADPH-oxidase-generated oxygen radicals, IC-mediated joint inflammation was significantly enhanced in p47phox^-/- ^mice. This might be due to a disruption in IC clearance because the removal of ICs from the joint determines the severity of arthritis [28]. This is in line with a previous study in which it was shown that oxygen radicals are crucial in the clearance of foreign particles such as cell walls of microorganisms [29]. Previously we found that injecting zymosan directly into the knee joint of p47phox^-/- ^mice caused a strongly elevated joint inflammation due to retarded clearance and resulted in prominent granuloma formation within the synovia of these mice [30]. In the present study we found that IFN-γ overexpression in the knee joint of p47phox^-/- ^mice before ICA induction prevented the increase in joint inflammation, and no granuloma formation was found. IFN-γ is a potent upregulator of receptors involved in phagocytosis, such as FcγR and complement receptors, and might lead to an efficient removal of the small amount of ICs responsible for continuing arthritis within the knee joints of p47phox^-/- ^mice. Macrophages form the dominant cell type within this model and these cells express large quantities of FcγR, largely responsible for IC clearance but also for the activation of the lining cells, driving arthritis [31]. Interestingly, synovial expression of the inhibitory FcγRII, which has been shown to be the dominant FcγR involved in IC clearance [32], was upregulated in the synovium of IFN-γ-stimulated p47phox^-/- ^mice, and because FcγRII does not need oxygen radicals for efficient clearance this might lead to a more efficient IC clearance.

Although the amount of infiltrated macrophages was not different between arthritic p47phox^-/- ^mice and their WT controls, destruction of the cartilage matrix by MMPs was lower in the absence of oxygen radicals. Cytokines such as IL-1 and TNFα activate chondrocyte and synoviocytes to produce MMPs, which are released in an inactive form. These latent enzymes need an activation step to become able to degrade the cartilage matrix. MMP-3 is the crucial MMP involved in the activation of MMP-13, which forms the rate-limiting enzyme in the degradation of the collagen type II matrix, leading to erosion of the cartilage matrix [5]. IFN-γ overexpression strongly increased MMP expression both in cartilage layers and in the synovium. This might be regulated directly by IFN-γ or indirectly in the synovium by the upregulation of FcγR and their subsequent activation by ICs. In the present study we found that inflamed synovia of IFN-γ-stimulated p47phox^-/- ^mice showed a strong upregulation of various MMPs such as MMP-9, MMP-12 and MMP-13, whereas only a minor upregulation of MMP-3 and MMP-12 was found within the cartilage. In the synovium, only TIMP-1 and TIMP-2 were marginally upregulated, whereas in the cartilage no differences in TIMP expression were found. Because MMP-mediated cartilage destruction was lower in arthritic p47phox^-/- ^mice, whereas MMP expression in the synovium and cartilage layers seemed higher, this might indicate that oxygen radicals, apart from inhibiting the gene expression of MMPs, are involved in their activation. Oxygen radicals have previously been shown to activate latent MMPs such as MMP-2 [17]. In the present study we also found that oxygen radicals upregulate FcγRI. Binding of ICs to FcγRI leads to more oxygen radical production [14] and might form an amplification step in the activation of pro-MMPs.

An interesting difference in the contribution of oxygen radicals to MMP-mediated cartilage damage in p47phox^-/- ^mice was found between arthritis induced by zymosan (ZIA) and that by ICs. During IFN-γ-stimulated ICA, oxygen radicals enhance MMP-cartilage damage, whereas during ZIA they inhibit it. An explanation for this discrepancy might be the cell type involved in mediating cartilage destruction. During ZIA, many polymorphonuclear cells (PMNs) infiltrate into the joint. Crucial enzymes released by PMNs are elastase and cathepsin G, which because of their highly positive charge are highly capable of penetrating cartilage and are then able to stimulate pro-MMPs into their active form, to generate VDIPEN neoepitopes [33]. Under normal circumstances elastase activity is inhibited by synovial fluid inhibitors such as α_2_-macroglobulin, and no VDIPEN staining can be detected within the cartilage layers [4]. However, in the absence of oxygen radicals the number of infiltrated PMNs was strongly increased during ZIA [30] and the amount of elastase might then overrule the inhibiting capacity of the synovial fluid.

**Figure 10:**
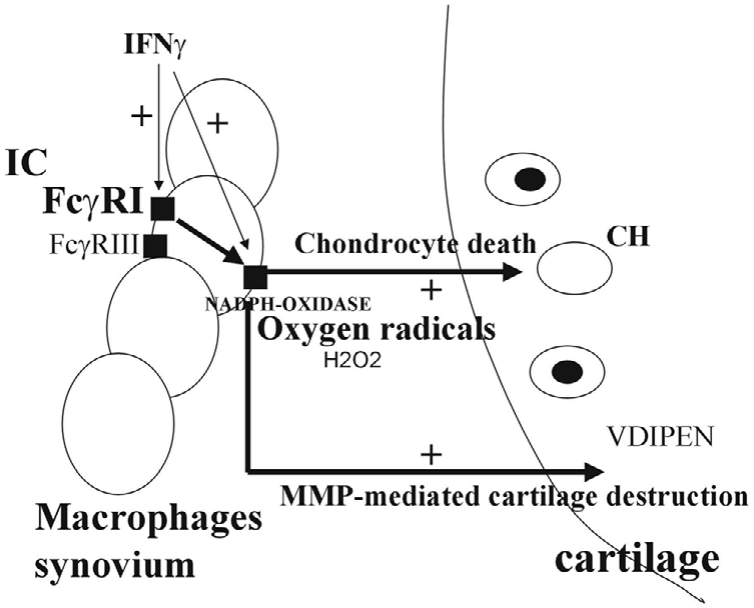
Possible involvement of NADPH oxidase in mediating cartilage destruction during ICA. Immune complexes (ICs) bind to FcγRI and FcγRIII expressed on macrophages, and cause the activation of NADPH oxidase. IFN-γ strongly elevates the expression of mainly FcγRI and NADPH oxidase. Oxygen radical production causes chondrocyte (CH) death and the activation of matrix metalloproteinases (MMP), leading to matrix degradation in the cartilage expressed as VDIPEN epitopes.

In contrast to ZIA, during IFN-γ-stimulated ICA the dominant infiltrating cell is the macrophage, which strongly attaches to the surface of the cartilage. The production of oxygen radicals such as hydrogen peroxide generated after the stimulation of FcγR by ICs [14] and the presence of superoxide dismutase might then be of crucial importance in regulating the activation of pro-MMPs in the cartilage matrix (Fig. 10). Hydrogen peroxide has a relatively long half-life and is able to activate pro-MMPs [17]. Synovial fluid contains large amounts of inhibitors of hydrogen peroxide such as catalase [34]. However, because of the close proximity of the activated macrophage to the cartilage surface, hydrogen peroxide can escape from this inhibitor, which owing to its large size (240 kDa) is not able to penetrate into the cartilage matrix [35].

Another parameter of severe cartilage destruction is chondrocyte death, which was completely abrogated in the absence of NADPH-oxidase-driven oxygen radicals. Chondrocyte death might be mediated by oxygen radicals released by the chondrocyte itself or by the inflamed synovium. Chondrocytes do express NADPH oxidase [36] and cytokines such as IL-1 are potent inducers of oxygen radicals in chondrocytes [37]. The production of intracellular hydrogen peroxide inside the chondrocyte can cause disruption of the mitochondrial membrane, leading to apoptosis [38]. However, earlier studies in our laboratory showed that FcγR activated synovium is of crucial importance in mediating chondrocyte death [39]. During IFN-γ-accelerated ICA, the infiltrated macrophages become activated by ICs, mainly via FcγR. In the mouse knee joint, FcγRI is expressed not by chondrocytes but exclusively by macrophages (and not neutrophils) and becomes strongly upregulated by IFN-γ. Binding of ICs to FcγRI in particular and, to a lesser extent, to FcγRIII leads to the activation of oxygen radical production (Fig. 10). Apart from FcγRI stimulation, IFN-γ itself has been shown to upregulate the p91 and p47 components of the NADPH oxidase and might contribute to the enhanced superoxide generation [40].

p47phox^-/- ^mice might also produce oxygen radicals by pathways other than the NADPH oxidase pathway [23]. However, IFN-γ alone had no effect on chondrocyte death. Moreover, it has been shown that IFN-γ does not upregulate alternative ways of oxygen radical production in p47phox^-/- ^mice [23]. This indicates that chondrocyte death is completely mediated via NADPH oxidase. IFN-γ induces the upregulation of NADPH oxidase components and FcγRI [41]. Stimulation of FcγRI by ICs might also lead to an enormous increase in oxygen radical production, mediating cartilage destruction (Fig. 10). Hydrogen peroxide might again be the most plausible oxygen species mediating chondrocyte death. Hydrogen peroxide can easily penetrate through cell membranes. Previous studies have shown that hydrogen peroxide, when injected into mouse knee joints, was able to induce considerable chondrocyte death, which might be induced by apoptosis [42]. Hydrogen peroxide activates the opening of the mitochondrial permeability transition pore and the release of cytochrome *c *[43]. In the cytoplasm, cytochrome *c*, in combination with Apaf-1, activates caspase-9, leading to the activation of caspase-3 and subsequent apoptosis [44].

NADPH oxidase and p47phox phosphorylation is strongly increased in leucocytes derived from synovial fluid of RA patients [45]. Cytokines such as IFN-γ are potent candidates for the upregulation of NADPH oxidase [41]. Moreover, ICs are found in considerable amounts in joints of many RA patients. These ICs might be responsible for a large part of NADPH oxidase activation via FcγRI stimulation, resulting in large quantities of oxygen radicals. The latter might mediate part of the severe cartilage destruction. Because FcγRI-mediated oxygen radical production might have a major function in mediating cartilage destruction during arthritis, this receptor might form a crucial target in combating this crippling disease.

## Conclusion

FcγR are central to the regulation of severe cartilage destruction during arthritis mediated by ICs. These ICs bind to FcγR, and the stimulation of activating FcγR, especially on synovial macrophages, leads to the production of as yet unknown products responsible for cartilage destruction. Th1 cytokines such as IFN-γ strongly upregulate FcγR – mainly FcγRI – and its stimulation leads to an enhanced production of oxygen radicals via NADPH oxidase. Using p47^-/- ^mice, which fail to produce oxygen radicals via NADPH oxidase, we have shown that during IFN-γ-stimulated IC-mediated arthritis, oxygen radicals completely determine chondrocyte death and aggravate MMP-mediated cartilage destruction. Blockade of signalling pathways regulating oxygen radical production via FcγR or by neutralising oxygen radicals directly may form new therapeutic methods of preventing severe cartilage destruction.

## Abbreviations

IC = immune complex; ICA = immune complex arthritis; IFN-γ = interferon-γ; IL = interleukin; MMP = matrix metalloproteinase; PG = proteoglycan; PMN = polymorphonuclear cells; RA = rheumatoid arthritis; RT-PCR = reverse transcriptase polymerase chain reaction; TIMP = tissue inhibitor of metalloproteinase; WT = wild-type; ZIA = zymosan-induced arthritis.

## Competing interests

The author(s) declare that they have no competing interests.

## Authors' contributions

PLEMvL conceived of the study, organised its design and coordination and drafted the manuscript. KCAMN participated in coordination, drafting of the manuscript, and performing the *in vivo *experiments and quantitative RT-PCR. ABB participated in coordination. AS performed the histology. AEMH performed the *in vivo *experiments. JK manufactured the adenoviral IFN-γ adenoviral construct. FAJVDL participated in drafting the manuscript. SMH manufactured the P47^-/- ^mice. WBVDB participated in drafting the manuscript. All authors read and approved the final manuscript.
